# Bridging the Gap between Evidence and Practice: Nationwide Retrospective Analysis of Lipid-Modifying Therapy Prescription Patterns in 5 Million Patients with Type 2 Diabetes Mellitus

**DOI:** 10.71079/aside.im.08252522

**Published:** 2025-08-25

**Authors:** Ahmed Hassan, Menna A. Keshk, Mohamed Reyad, Nourhan Ahmed, Omar Nassar, Aisha Siraj, Salem Badr, Sherif Eltawansy, Anoop Misra, Muhammed Amir Essibayi, Ahmed Y. Azzam, Mahmoud Nassar, Diaa Hakim

**Affiliations:** 1- Department of Cardiology, Suez Medical Complex, Ministry of Health and Population, Suez, Egypt; 2- Faculty of Medicine, Cairo University, Cairo, Egypt; 3- Cardiology Department, Banner University Medical Center, Phoenix, AZ, United States; 4- Department of Nephrology, Suez Medical Complex, Ministry of Health and Population, Suez, Egypt; 5- Champlain Valley Union High School, Hinesburg, VT, United States; 6- MetroHealth Medical Center, Case Western Reserve University, Ohio, United States; 7- Aurora Cardiovascular Services, Aurora Sinai/Aurora St. Luke’s Medical Centers, Milwaukee, WI, United States; 8- Internal Medicine Department, Jersey Shore University Medical Center, Neptune, NJ, United States; 9- Chairman, Fortis-C-DOC Centre of Excellence for Diabetes, Metabolic Diseases and Endocrinology, Chirag Enclave, New Delhi, India; 9- Chairman, National Diabetes, Obesity and Cholesterol Foundation (N-DOC), SDA, New Delhi, India; 9- President, Diabetes Foundation (India) (DFI), Vasant Kunj, New Delhi, India; 10- Montefiore-Einstein Cerebrovascular Research Lab, Montefiore Medical Center, Albert Einstein College of Medicine, Bronx, NY, United States; 11- SNU Medical Big Data Research Center, Seoul National University, Gwanak-gu, Seoul, South Korea; 12- Division of Endocrinology and Diabetes, Larner College of Medicine, University of Vermont, Burlington, VT, United States; 13- Cardiovascular Medicine Division, Brigham and Women’s Hospital, Harvard Medical School, Boston, MA, United States

**Keywords:** Diabetes Mellitus, Cardiovascular Disease, Statin, Lipid-Modifying Drugs, Retrospective observational analysis

## Abstract

**Introduction:**

Type 2 diabetes mellitus (T2DM) is associated with dyslipidemia and significantly increased cardiovascular risk, making lipid-modifying therapy a crucial preventive intervention in these patients. Despite clear guidelines recommending statin therapy for both primary and secondary prevention, real-world prescription routines and practices show gaps in clinical care. We aimed to evaluate the rates and patterns of lipid-modifying therapy under prescription among T2DM patients across U.S. healthcare facilities.

**Methods:**

We conducted a retrospective observational analysis using the TriNetX US Collaborative Network database, including data from 69 healthcare organizations throughout the United States. Patients with T2DM patients aged 40–75 years were included in our cohort. Under-prescription rates were calculated and analyzed across demographic subgroups using standardized protocols within the TriNetX platform.

**Results:**

Among 5,007,910 T2DM patients, we observed significant statin under-prescription rates. Our analysis showed a prescription rate of 55.1% for statins in eligible patients with T2DM.

**Conclusions:**

Our findings revealed a significant under-prescription of lipid-modifying therapy in T2DM patients. The universal nature of under-prescription suggests barriers to guideline implementation. These results underscore the urgent need for systematic interventions, including automated identification systems, standardized protocols, and optimized provider education to improve cardiovascular risk management in patients with T2DM.

## 1. Introduction

Cardiovascular disease remains the leading cause of morbidity and mortality among patients with type 2 diabetes mellitus (T2DM), accounting for approximately 50% of all deaths in this population [1]. The relationship between T2DM and cardiovascular complications is well-established, with diabetic patients experiencing a two to four-fold increased risk of cardiovascular events compared to non-diabetic patients [2]. This heightened risk underscores the importance of preventive strategies, especially lipid-modifying therapy, in reducing cardiovascular events among T2DM patients [2]. Weight loss and lifestyle modification are cornerstone strategies for managing T2DM, contributing to improved glycemic control, enhanced insulin sensitivity, and reduced cardiovascular risk. In addition to these foundational measures, statins and other lipid-modifying agents represent cornerstone therapeutic interventions in T2DM management, with clinical evidence supporting their role in both primary and secondary cardiovascular prevention [3, 4, 5].

Current clinical guidelines strongly recommend statin therapy for T2DM patients aged 40–75 years [6]. Despite these clear recommendations and the proven benefits of lipid-modifying therapy, mounting evidence suggests significant gaps between guideline recommendations and real-world prescription practices and routines [3, 4, 5]. The underutilization and under-prescription of statins and other lipid-modifying agents in high-risk populations represents a significant healthcare quality gap that may contribute to preventable cardiovascular events [7]. Previous studies have reported varying rates of statin underuse, ranging from 30% to 60% among eligible patients. However, precise analyses of prescription practices across different risk categories and demographic groups remain limited, particularly in large, diverse patient populations [8, 7]. Understanding the extent and patterns of lipid-modifying therapy prescribing patterns is crucial for developing targeted interventions to improve guideline adherence and patient outcomes [8, 7]. In addition to that, identifying possible disparities in prescription practices across different demographic groups could help address systemic barriers to better cardiovascular care in T2DM patients. We aimed to evaluate statin and lipid-modifying agent prescriptions among T2DM patients aged 40–75 years using a large U.S. multi-institutional database. Our primary focus was quantifying under-prescription rates and investigating demographics influencing prescribing patterns.

## 2. Methods

Our investigation employed a retrospective observational approach using the TriNetX US Collaborative Network, aggregating deidentified electronic health records from over 69 healthcare organizations across the United States. This network encompasses academic medical centers, community hospitals, and outpatient clinics, providing a diverse patient population that enhances the generalizability of our findings. All data extraction complied with HIPAA regulations and institutional privacy requirements, with records available up to December 14, 2024, and no lower date boundary.

### 2.1. Outcome and Definition

We defined our primary outcome as the under-prescription rate of lipid-modifying therapy among patients with T2DM aged 40–75 years. Under-prescription was operationalized as the proportion of eligible patients without any statin or alternative lipid-modifying agent prescription, identified through standardized ATC codes (C10AA, C10, and C10A) within the database. The diagnostic criteria for T2DM and treatment eligibility were established using ICD-10 codes, laboratory values, and clinical documentation.

### 2.2. Study Design

**Figure 1:**
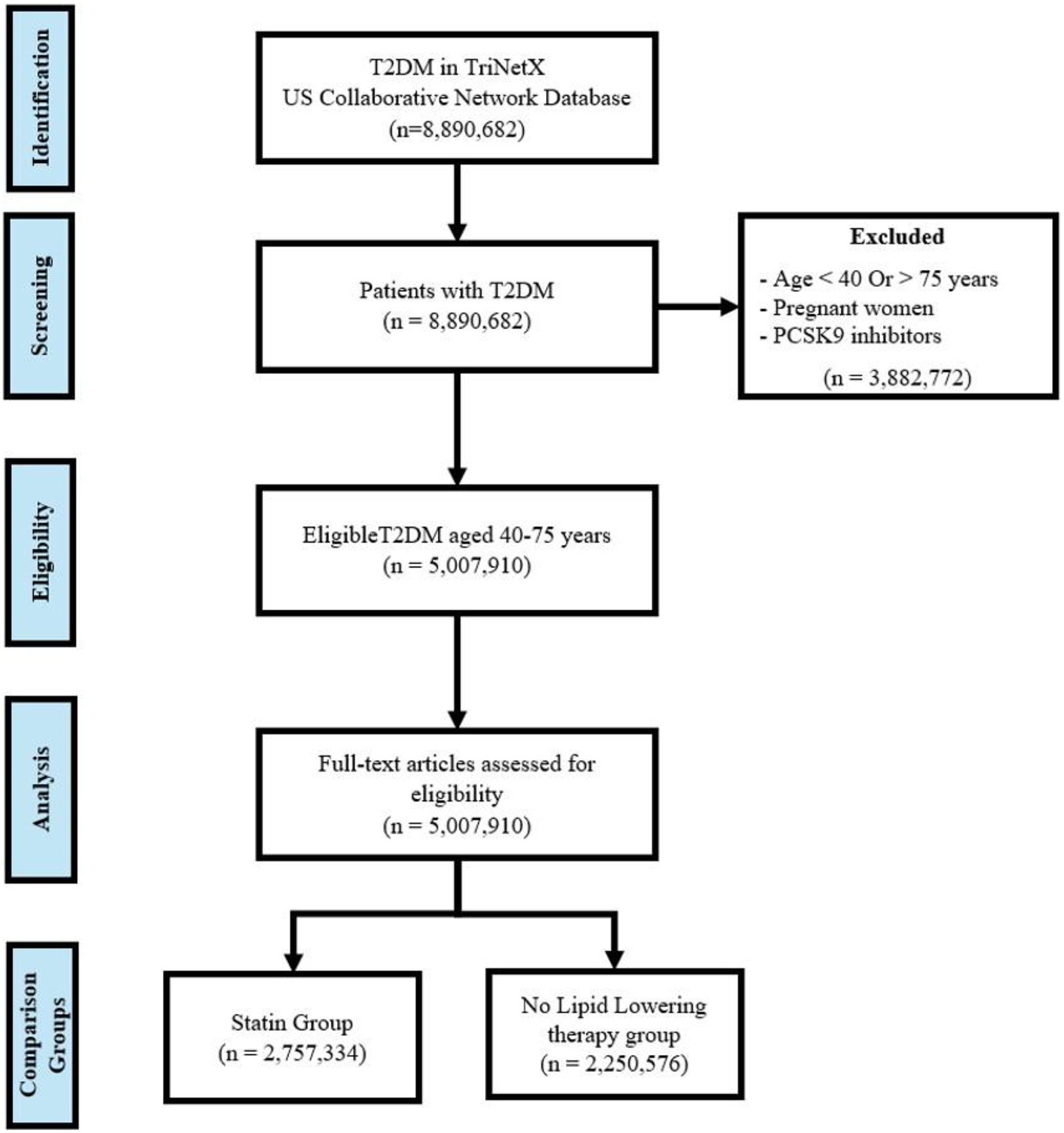
Selection Process and Classification of Patients with T2DM for Statin Under-Prescription Analysis.

This retrospective analysis focused specifically on T2DM patients aged 40–75 years—a population for whom clinical guidelines recommend statin therapy. We excluded non-diabetic individuals and patients with diabetes who are younger than 40 years or older than 75 years. Also, we excluded pregnant patients due to contraindications for statin therapy during pregnancy and potential confounding effects on lipid metabolism. Additionally, patients receiving PCSK9 inhibitors were excluded from the analysis, as these medications represent an alternative lipid-lowering strategy typically reserved for patients with statin intolerance or inadequate response to maximum statin therapy, and thus would have introduced heterogeneity in our assessment of guideline-directed statin therapy implementation This study specifically focused on analyzing the prescription patterns of statins as the primary recommended lipid-modifying therapy for T2DM patients according to current guidelines (Figure 1).

Patient selection utilized structured query language protocols within the TriNetX platform to identify individuals meeting our inclusion criteria. For medication exposure assessment, we employed anatomical therapeutic chemical codes, specifically C10AA (HMG CoA reductase inhibitors), and the broader categories C10 and C10A (lipid-modifying agents), to ensure comprehensive capture of all relevant treatments. This approach minimized the risk of missing prescriptions due to varying coding practices across healthcare organizations. The study’s generalizability was strengthened by including a large, geographically diverse patient sample, while potential biases were reduced through validated algorithms for disease diagnosis and medication exposure.

### 2.3. Quality Assurance

We implemented rigorous quality assurance throughout our analysis, employing validated algorithms for T2DM diagnosis confirmation that incorporated both ICD-10 codes and supporting clinical documentation. Cardiovascular event history underwent multi-phased verification through diagnostic codes, procedure records, and clinical notes review. Laboratory data quality was maintained through standardized value range validation and unit conversion protocols. The TriNetX platform automatically detected missing data across all variables. No manual imputation was performed; we relied on the platform’s data integrity checks to ensure comprehensive and accurate cohort definitions.

### 2.4. Statistical Analysis

Demographic analysis included extraction of age and gender. We analyzed continuous variables using descriptive statistics (means, standard deviations, ranges) and categorical variables using frequencies and percentages. Under-prescription rates were calculated as the proportion of eligible patients without lipid-modifying therapy prescriptions. For comparative analyses, we employed chi-square tests of independence to evaluate differences in statin prescription rates between categorical demographic subgroups (e.g., gender). For continuous variables (e.g., age), independent samples t-tests were used to compare differences between the statin and non-statin groups. Statistical significance was set at p<0.05, though with our large sample size, we considered both statistical significance and clinical relevance when interpreting results. Statistical analyses utilized the integrated tools within the TriNetX platform. This protocol received an exemption from institutional review board approval due to the use of de-identified data, though we maintained strict adherence to ethical research principles throughout. Our analysis complied with STROBE guidelines for observational studies [9] and RECORD statement recommendations for studies utilizing routinely collected health data [10].

## 3. Results

### 3.1. Baseline Characteristics and Prescription Patterns

Our cohort included 5,007,910 patients with T2DM aged 40– 75 years. Of these, 2,757,334 (55.1%) were prescribed statins, while 2,250,576 (44.9%) received no statin or other lipid-lowering therapy. The statin group had a higher mean age (63 ± 9 years) compared to the non-statin group (60 ± 10 years), suggesting that statin prescription rates increase with advancing age **??**.

### 3.2. Gender-Based Prescription Patterns

Analysis of prescription patterns by gender revealed notable disparities. Male patients had higher statin prescription rates (57.5%) than female patients (52.8%), representing a 4.7% difference. Among the statin group, males constituted 51.75% (n=1,426,920) of patients, while females accounted for 45.48% (n=1,254,035). Conversely, in the non-statin group, females represented a higher proportion (49.87%, n=1,122,362) than males (46.82%, n=1,053,920). For patients with gender not reported or not specified, the prescription rate was 50.7%, falling between the rates for males and females (Table 1).

### 3.3. Overall Prescription Rate

The overall statin prescription rate of 55.1% indicates that nearly half of eligible patients with T2DM aged 40–75 years were not receiving guideline-recommended statin therapy, highlighting a significant gap between clinical practice and guideline recommendations. This finding suggests substantial opportunities for improvement in cardiovascular risk management for patients with T2DM across healthcare organizations in the United States.

### 3.4. Data Completeness

Data for some variables were missing for 6.08% of participants, primarily affecting demographic information rather than prescription status.

## 4. Discussion

In this retrospective observational analysis, we investigated the lipid-lowering therapy prescriptions across 69 U.S. healthcare organizations and identified a statin prescription rate of 55.1% among T2DM patients aged 40–75 years. This substantial discrepancy between the current clinical practice and guideline recommendations emphasizes the ongoing systemic challenges in effectively managing cardiovascular risk in this high-risk cohort. Registry-based studies in developed nations have shown an increase in statin prescriptions for patients with type 2 diabetes following the endorsement of their use by the AHA/ACC guidelines [11]. Statin use among diabetes patients has been documented in several countries, such as the Swedish National Diabetes Register [12], the British National Health Service (NHS) [13], and Australian General Practices [14], where the rate of prescription for diabetes patients ranged from 60–70%. However, our study’s percentages are lower than those seen in Swedish, British, and Australian registries (10–12). In our study, 55.1% of patients were prescribed statins, similar to the 51% reported by the US National Health and Nutrition Examination Survey (NHANES)[15] for individuals with diabetes aged 40 and above. Despite this, the overall prescription rates for statins, including high-dose statins, remain suboptimal and are considerably below the guidelines. A previous study by Johansen et al. [16] suggested that the reason for undertreatment is the focus on the hyperlipidemia profile rather than overall cardiovascular risk. Many individuals at risk of cardiovascular events, including those with diabetes, were not receiving statins as a tool to reduce cardiovascular disease (CVD) risk. This suggests that statin use is strongly driven by the hyperlipidemia profile rather than overall cardiovascular risk. Although the 2013 AHA guidelines [11] recommended that all patients with diabetes receive high-dose statins, irrespective of cholesterol levels, a study by Pokharel et al. [17] In 2016, it showed that 38% of patients with diabetes who did not have CVD had no documented statin prescription. Providers might be reluctant to start statin therapy for primary prevention in diabetic patients without overt cardiovascular disease [4?]. This overdependence on cholesterol levels indicates that individuals with hyperlipidemia but without diabetes or heart disease are more likely to be prescribed statins than those without hyperlipidemia who have diabetes or heart disease[18]. To address this, the Cholesterol Treatment Trialists’ Collaboration study recently showed that while statin therapy may have modest effects on glycemia, the cardiovascular benefits outweigh the potential risks in diabetic patients due to the aggregate effects of statins on blood lipids and glycemia, so even if statins cause a slight increase in glycemia, or have any other theoretical adverse effects, these are already offset by the overall reduction in cardiovascular risk observed with statin therapy. Additionally, the risk of future major vascular events is significantly higher following a major vascular event than following a diagnosis of diabetes [8]. Another possible reason for the lower use of statins may be inadequate documentation, such as insufficient records of statin intolerance [17]. An additional consideration is the universal nature of statin under-prescription across demographic groups. An interesting finding from our analysis is supported by previous studies that identified demographic variations in statin use. We found significant differences in prescription patterns based on gender [8, 7]. This suggests that barriers to guideline implementation impact all patient populations, pointing to broader systemic issues in healthcare delivery rather than factors related to access or bias. Prescribing Statin to patients is economically efficient; a study by McConnachie et al. revealed that statin therapy decreases the rate of cardiovascular rehospitalization and the length of hospital stay, which results in less financial burden. Additionally, statin increases the patient’s quality of life [19]. The importance of our study lies in highlighting the urgent need for systematic interventions to improve guideline adherence in clinical practice. Identifying barriers to optimal statin use and implementing interventions, such as audit/feedback or decision support tools and precision medicine, are essential to bridging the gap between guidelines and practice. Given the above, our findings suggest that healthcare systems must implement multi-phasic strategies that address provider- and system-level barriers to appropriate statin prescribing. These strategies should include automated identification systems for eligible patients, standardized prescription protocols, regular monitoring of quality metrics, enhanced provider education programs, and integrated clinical decision support tools. Additionally, raising awareness among healthcare providers that diabetic patients may benefit from statin therapy regardless of their LDL levels. Shifting the focus from hyperlipidemia to overall cardiovascular risk could lead to better outcomes. Our study has several important limitations. As a retrospective analysis, we couldn’t capture all factors influencing prescribing decisions, such as patient preferences, medication intolerance, or undocumented contraindications. We focused solely on prescription data without assessing medication adherence or analyzing prescription patterns by provider specialty, which may significantly impact practices. Without LDL-C data or comprehensive cardiovascular risk profiles, we cannot determine whether prescribing patterns correlate with clinical risk assessments. Our analysis didn’t assess statin intensity despite guidelines recommending moderate to high-intensity statins for T2DM patients, nor did we conduct temporal trend analysis to evaluate improvements over time. While we identified gender disparities, we didn’t thoroughly analyze other demographic factors like race/ethnicity or socioeconomic status, or consider how comorbidities might influence prescribing decisions. We also focused exclusively on statins without examining alternative lipid-lowering agents, potentially overestimating gaps in lipid management. Additionally, while our database includes data from 69 healthcare organizations, the applicability of our results to other settings, particularly rural or underserved areas, requires further consideration. Future research should focus on understanding barriers to statin prescribing at both the provider and system levels. Qualitative studies on decision-making, patient perspectives, and prospective implementation studies could help identify effective approaches to improving prescription rates and cardiovascular risk management in the T2DM population.

## 5. Conclusion

In this retrospective analysis, we showed that a considerable number of diabetic patients—across different demographic groups — who would potentially benefit from statins were not receiving this therapy. Our findings indicate the need for system-level interventions and warrant a call for emergent action from healthcare providers and organizations to address the current care gaps. This change could lead to substantial societal benefits.

## Figures and Tables

**Table 1:** Baseline Characteristics and Statin Prescription Rates Among Patients with Type 2 Diabetes Mellitus Aged 40–75 Years (N=5,007,910)

Characteristic	Statin Group (n=2,757,334)	No Statin/Other Therapy (n=2,250,576)	P-value
**Age, years**			
Mean ± SD	63 ± 9	60 ± 10	<0.001
**Sex, n (%)**			
Male	1,426,920 (51.75%)	1,053,920 (46.82%)	<0.001
Female	1,254,035 (45.48%)	1,122,362 (49.87%)	
Not Reported / Not Specified	76,378 (2.77%)	74,294 (3.31%)	
**Total**	2,757,334 (100%)	2,250,576 (100%)	-
**Prescription Rate**	57.5% (Male)	52.8% (Female), 50.7% (NR/NS)	-
**Overall Prescription Rate**		55.1%	

Values for age are presented as mean ± standard deviation (SD) and range. Sex and prescription rates are presented as absolute numbers (n) with percentages (%) in parentheses.

## Data Availability

Available on TriNetX Database Based on Institutional Collaborations.
